# PARP inhibitor shuts down the global translation of thyroid cancer through promoting Pol II binding to DIMT1 pause

**DOI:** 10.7150/ijbs.81895

**Published:** 2023-07-31

**Authors:** Xiukun Hou, Mengran Tian, Junya Ning, Zhongyu Wang, Fengli Guo, Wei Zhang, Linfei Hu, Songfeng Wei, Chuanxiang Hu, Xinwei Yun, Jingzhu Zhao, Qiman Dong, Xianhui Ruan, Dapeng Li, Ming Gao, Xiangqian Zheng

**Affiliations:** 1 Department of Thyroid and Neck Cancer, Tianjin Medical University Cancer Institute and Hospital, National Clinical Research Center for Cancer, Key Laboratory of Cancer Prevention and Therapy, Tianjin's Clinical Research Center for Cancer, Tianjin, 300040, China.; 2 Department of Thyroid and Breast Surgery, Tianjin Union Medical Center, Tianjin, 300121, China.; 3 Tianjin Key Laboratory of General Surgery in Construction, Tianjin Union Medical Center, Tianjin,300121, China.; 4 School of Medicine, Nankai University, Tianjin 300071, China.; 5 State Key Laboratory of Medicinal Chemical Biology, Key Laboratory of Bioactive Materials, Ministry of Education, Tianjin Key Laboratory of Protein Sciences and College of Life Sciences, Nankai University, Tianjin 300071, China.

**Keywords:** PARP inhibitor, BRCAness genes, Pol II pausing, NF-κB signaling pathway, combination treatment

## Abstract

Thyroid cancer has become the most frequent endocrine-related malignancy. Currently, a mounting body of evidences support the clinical strategies for extending the benefit of PARP inhibitors beyond BRCA-mutant cancers. However, the functions and molecular mechanisms of PARP inhibitors in thyroid cancers (TCs) are not fully understood. Here, on the one hand, we revealed that niraparib promotes the accumulation of DNA damage in TCs. On the other hand, we indicated that niraparib inhibits the transcription of DIMT1 through promoting Pol II pausing in a PAR-dependent manner, subsequently leading to a global translation inhibition in TCs. Meanwhile, we found that niraparib activates the NF-κB signaling pathway by inhibiting the PARylation of p65, which decreases its ubiquitination and degradation level through E3 ubiquitin ligase RNF146. Moreover, bortezomib (a small molecule inhibitor of the NF-κB signaling pathway) could significantly enhance the anti-tumor effect of niraparib on TCs in vitro and in vivo. Our findings provide mechanistic supports for the efficacy of PARP inhibitors in cancer cells lacking BRCA-mutant.

## Introduction

The incidence of thyroid cancer has dramatically increased during the past decades. Up to now, TC has become the most frequent endocrine-related malignancy 1. Based on the histopathological features, TC has been clarified into different subtypes, including papillary thyroid carcinoma (PTC) and anaplastic thyroid carcinoma (ATC). Although the majority of PTCs display a good prognosis, still approximately 15% of them occurred recurrence and with a poor prognosis 2. Meanwhile, ATCs are highly aggressive and always resistant to conventional treatment including radioactive iodine and chemotherapy. Over 90% of ATCs lost the opportunity of surgery at the time of diagnosis and dead in less than one more year 3. Therefore, searching for a new treatment strategy for TCs is of great significance.

Poly(ADP-ribose) polymerase synthesis poly (ADP-ribose) (PAR) chains on target proteins as a post-translational modification is involved in multiple biological processes including DNA damage response, chromatin structure reorganization, and transcription regulation 4. Synthetic lethal interaction between PARP inhibition and BRCA gene mutation has been described in 2005 5, 6. By now, there have already several PARP inhibitors been approved for the treatment of BRCA1/2 mutated breast, ovarian, pancreatic, and prostate cancers 7-10. During the past decades, the understanding of the biological functions of BRCA gene has made considerable progress. BRCAness genes, including PIK3CA, TP53, CDKN2A, PTEN, etc which can lead to homologous recombination repair deficiency (HRD) have been proposed 11. Research revealed the mutational landscape of TCs and found that a reasonable quantity of BRCAness genes mutated in TCs 12. This prompted us to investigate whether PARP inhibitors could be the therapeutic approach for TCs.

Ribosome biogenesis includes a series of organized steps that participate in protein synthesis 13. Among these steps, RNA polymerase II (Pol II) mediates some ribosomal gene transcription to produce the large pre-60S and the small pre-40S pre-ribosomal subunits 14. The progression of cancer cells is associated with an increase in ribosome biogenesis and therefore represents an attractive therapy target 15. Studies revealed that BRCA1/2-intact breast cancer cells treated with PARP inhibitors reduce ribosome biogenesis and cell growth 16. But the molecular mechanism between PARP inhibitors and protein synthesis is still unclear.

The NF-κB signaling pathway is involved in tumor development and progression and plays key roles in tumor survival, growth, and relapse 17, 18. The activation of the NF-κB pathway has been reported in response to a variety of anti-cancer therapeutic agents and its inhibition has been observed to re-sensitize drug-resistant cells to anti-cancer agents 19, 20. Researches showed that PARP inhibitors activate the NF-κB signaling pathway and its inhibitor enhanced the anti-cancer efficiency of PARP inhibitors in ovarian cancer 21, 22. However, the molecular mechanisms of how PARP inhibitors activate the NF-κB signaling pathway have not yet been investigated.

In this research, apart from the classical function of niraparib that promotes the accumulation of DNA damage in TCs, we also demonstrated, on the one hand, that niraparib inhibits the transcription of DIMT1 through promoting Pol II pausing in a PAR-dependent manner, subsequently leading to a global translation inhibition in TCs. On the other hand, we found that niraparib activates the NF-κB signaling pathway by inhibiting the PARylation of p65, which decreases its ubiquitination and degradation level through E3 ubiquitin ligase RNF146. Moreover, bortezomib (a small molecule inhibitor of the NF-κB signaling pathway) could significantly enhance the anti-tumor effect of niraparib on TCs *in vitro* and *in vivo*. Our findings provide mechanistic supports for the efficacy of PARP inhibitors in cancer cells lacking BRCA-mutant.

## Methods

### Cell culture, transfection, and lentiviral infection

Four thyroid cancer cell lines (Cal-62, 8305C, B-CPAP, TPC-1,) and one HEK293T cell line were included in this research. All the cell lines were cultured in RPMI-1640 medium or DMEM (Gibco, USA) supplemented with 10% fetal bovine serum (FBS) and 1% penicillin and streptomycin and maintained in a humidified atmosphere with 5% CO2 at 37°C. Small interfering RNA (siRNA) targeting PARP1 and RNF146 were transfected with Lipofectamine 2000 (Invitrogen) according to the manufacturer's protocol. The sequences of siRNAs are listed in Supplementary Table 1.

Lentiviral particles were produced in HEK293T cells by transient co-transfection of transfer vector constructs (pLKO.1-Puro vectors), VSVg, and Delta 8.9. Transfection of HEK293T cells was performed using Lipofectamine 2000 (Invitrogen) according to the manufacturer's protocol.

### Reagents, drugs, and antibodies

The following primary antibodies were used in the research: anti-Ki67, anti-phospho-γH2A, anti-DIMT1, anti-GAPDH, anti-NELF-E, anti-PARP1, anti-p65, anti-phospho-p65, anti-ubiquitin, anti-p21, (Cell Signaling Technology, USA). Anti-puromycin, anti-poly-ADP-ribose, (Merck Millipore, USA). Anti-RNA polymerase II, anti-RNA polymerase II (phospho-S5), anti-RNA polymerase II (phospho-S2), anti-RNF146, (Abcam, USA). Anti-PARP1 (Active Motif, USA). Niraparib, PJ34, olaparib, and rucaparib were purchased from Selleck Chemicals (Houston, USA). Talazoparib was purchased from TargetMol (Boston, USA). 5,6-Dichlorobenzimidazole 1-β-D-ribofuranosylbenzimidazole (DRB) was purchased from APExBIO (Houston, USA).

### Immunohistochemistry (IHC)

The tissue samples of the tumors were subjected to immunohistochemical staining according to a standard protocol 23. The signal was visualized with the DAB Substrate Kit (MaiXin Bio, China). For Ki-67 staining, positively stained cells were counted in five files and the total number of positive cells was calculated.

### Cell viability and colony formation assay

A total of 1000-1500 cells were seeded per well of 96-well plates. Next, cell viability was assessed with Cell Counting Kit-8 (CCK-8) according to the protocol. For colony formation assays, 500-1000 cells per well were seeded in six-well plates and cultured for 1-2 weeks. Then, colonies were fixed with 100% methanol for 20 min and stained with 0.5% crystal violet for 15 min. The cells were treated with niraparib (5 uM) and/or bortezomib (5 nM).

### RNAseq processing

Total RNA was extracted from the Cal-62 cells treated with DMSO or niraparib. RNA-Seq reads were trimmed using Trim Galore (v0.5.0). The trimmed data were aligned to the human hg19 genome using STAR (v2.7.0 f), and the aligned bam files were sorted by name using the parameter-n. Sequencing data were deposited in the Gene Expression Omnibus (GSE217620).

### Gene Ontology (GO) analysis, KEGG pathway analysis, and Gene Set Enrichment Analysis (GSEA)

The Database for Annotation, Visualization, and Integrated Discovery (DAVID) web tool was used to perform GO analysis and KEGG pathway analysis. Significant enrichment was defined as p < 0.05. GSEA was performed with the GSEA stand-alone desktop program. Significantly enriched molecular function terms were defined by p < 0.05.

### Comet assay

The comet assay was performed according to the OxiSelect™ Comet Assay Kit (Cell Biolabs, San Diego) protocol. Cell samples with Comet Agarose (step 2) at 1:10 ratio (v/v) were transferred onto the OxiSelect™ Comet Slide. The slides were kept at 4ºC in the dark for 15-30 minutes. Then the slides were immersed in pre-chilled Lysis Buffer and pre-chilled Alkaline Solution for 30-60 minutes. The slides were equilibrated in an electrophoresis buffer and incubate with Vista Green DNA Dye for 15 minutes. Images were generated using Zeiss confocal microscope.

### TMT labeling and LC-MS/MS analysis

Proteins from cells were extracted with an appropriate amount of protein lysate (8 M urea, 1% SDS), which contains protease inhibitors to inhibit protease activity. The TMT reagent (Thermo Fisher, A44522) was added to 50 μg polypeptide and incubated for 2 h at room temperature.

Labeled peptides were analyzed by online nanoflow liquid chromatography-tandem mass spectrometry (LC/MS). The experiments were performed on a 9RKFSG2_NCS-3500R system (Thermo, USA) connected to Q_Exactive HF-X (Thermo, USA).

### Chromatin immunoprecipitation (ChIP)-Seq and data analysis

ChIP-seq of Cal-62 cells was prepared with SimpleChIP® Plus Enzymatic Chromatin IP Kit (Magnetic Beads) (Cell Signaling) according to the manufacturer's instructions. Immunoprecipitation was performed with a PARP1 antibody (Active Motif).

ChIP-Seq peaks were generated by the peak-finding algorithm model-based analysis for ChIP-Seq v1.4.2. IGV tools v2.4.5 was used to visualize the ChIP-Seq tracks. The total PARP1 ChIP-seq signal is expressed in units of RPM per bin. Homer was used to merge and stitch the peaks within 12.5 kb. Sequencing data were deposited in the Gene Expression Omnibus (GSE219292).

### ChIP-qPCR

ChIP was performed with the Simple Chip Enzymatic Chromatin IP Kit (Cell Signaling) according to the manufacturer's instructions. The primers for qPCR are listed in Supplementary Table 2.

### Immunofluorescence staining

Cells were seeded on 22 × 22 mm glass coverslips. Cultured cells were fixed using 4% paraformaldehyde. Samples were blocked with 5% normal goat serum with 0.2% Triton X-100 (Sigma-Aldrich) in PBS for 30 min at room temperature and were then incubated with primary antibodies overnight at 37°C, followed by the appropriate secondary fluorescently labeled antibodies (Invitrogen, 1:1000) for 1 hour at 37°C. Nuclei were counterstained with DAPI. Images were acquired using Zeiss confocal microscopy.

### Cell cycle analysis

In brief, thyroid cancer cells treated with DMSO or niraparib were fixed with 90% chilled ethanol overnight. Fixed cells were then washed and resuspended in phosphate-buffered saline (PBS) containing 1% normal goat serum (NGS) and then incubated with PBS containing 10 μg/ml of RNase A and 120 μg/ml of PI for 30 min in the dark at 37°C. Finally, Stained cells were measured with a FACSCalibur flow cytometer (BD Biosciences). The cells were treated with niraparib (5 uM) and/or bortezomib (5 nM) for 48 h.

### WB-SUnSET assay

Thyroid cancer cells treated with DMSO or niraparib were cultured with 10 µM puromycin for 10 min before harvest. Cells were washed twice with ice-cold PBS and the same number of cells were then lysed in 200 μL of cell lysis buffer. Then the samples were mixed with an equal volume of 2X SDS PAGE loading buffer and were submitted to western blot. Puromycin-labeled polypeptides were then quantified by incubating membranes with anti-puromycin.

### Total RNA extraction and quantitative real-time PCR

Total RNA was isolated by TRIzol reagent (Invitrogen, Carlsbad, USA), and cDNA was reverse transcribed with HiScript III RT SuperMix for qPCR kit (+gDNA wiper) (Vazyme, Nanjing, China). Quantitative real-time PCR was performed with the SYBR Premix Ex Taq II kit (Vazyme, Nanjing, China) and specific primers. The sequences of the primers are shown in Supplementary Table 3.

### Western blotting

Proteins from cells were extracted with radioimmunoprecipitation assay buffer (Solarbio, Beijing, China) according to the protocol. The concentration of protein samples was quantified by the BCA assay. Samples were run on 8-12% sodium dodecyl sulfate-polyacrylamide gel electrophoresis gels and transferred to polyvinylidene difluoride membranes. Then, the cells were incubated with primary antibodies and peroxidase-conjugated anti-mouse or anti-rabbit IgG antibodies. Finally, the cells were visualized with Sparkjade ECL super (Sparkjade, Shandong, China).

### Co-immunoprecipitation

For co-immunoprecipitation experiments, Cal-62 cells were collected and immunoprecipitated with 4 μg primary antibodies overnight at 4°C. Then the lysates were incubated with 50 μl protein G Dynabeads for 2h. The precipitants were washed extensively with wash buffer, boiled with SDS loading buffer, and subjected to SDS-PAGE and immunoblotting.

### Animal studies

All of the animal studies were approved by the Ethics Committee of the Tianjin Medical University Cancer Institute and Hospital and were carried out following the National Institutes of Health Guide for the Care and Use of Laboratory Animals. A total of 2 × 10^6^ Cal-62 cells were injected into 5-week-old female BALB/c nude mice. The mice were administered PARP inhibitors and/or bortezomib as described 24, 25.

Tumors were measured every two days. The tumor volume was calculated using the formula V = 0.5 × larger diameter × (smaller diameter)^2^. Then the mice were sacrificed, and the tumors were collected and analyzed by hematoxylin and eosin (H&E) staining. All of the mice were purchased from SPF Biotechnology (Beijing, China).

### Statistical analysis

All data were analyzed with GraphPad Prism Ver. 9.0 (CA, USA). The data are presented as the mean ± SD of at least three independent experiments. Two-way ANOVA was used to compare multiple groups. All data were analyzed with Student's t-test (***p < 0.001, **p < 0.01, *p < 0.05).

## Results

### PARP inhibitors block the growth of thyroid cancer cells *in vitro* and *in vivo*

**Figure 1:**
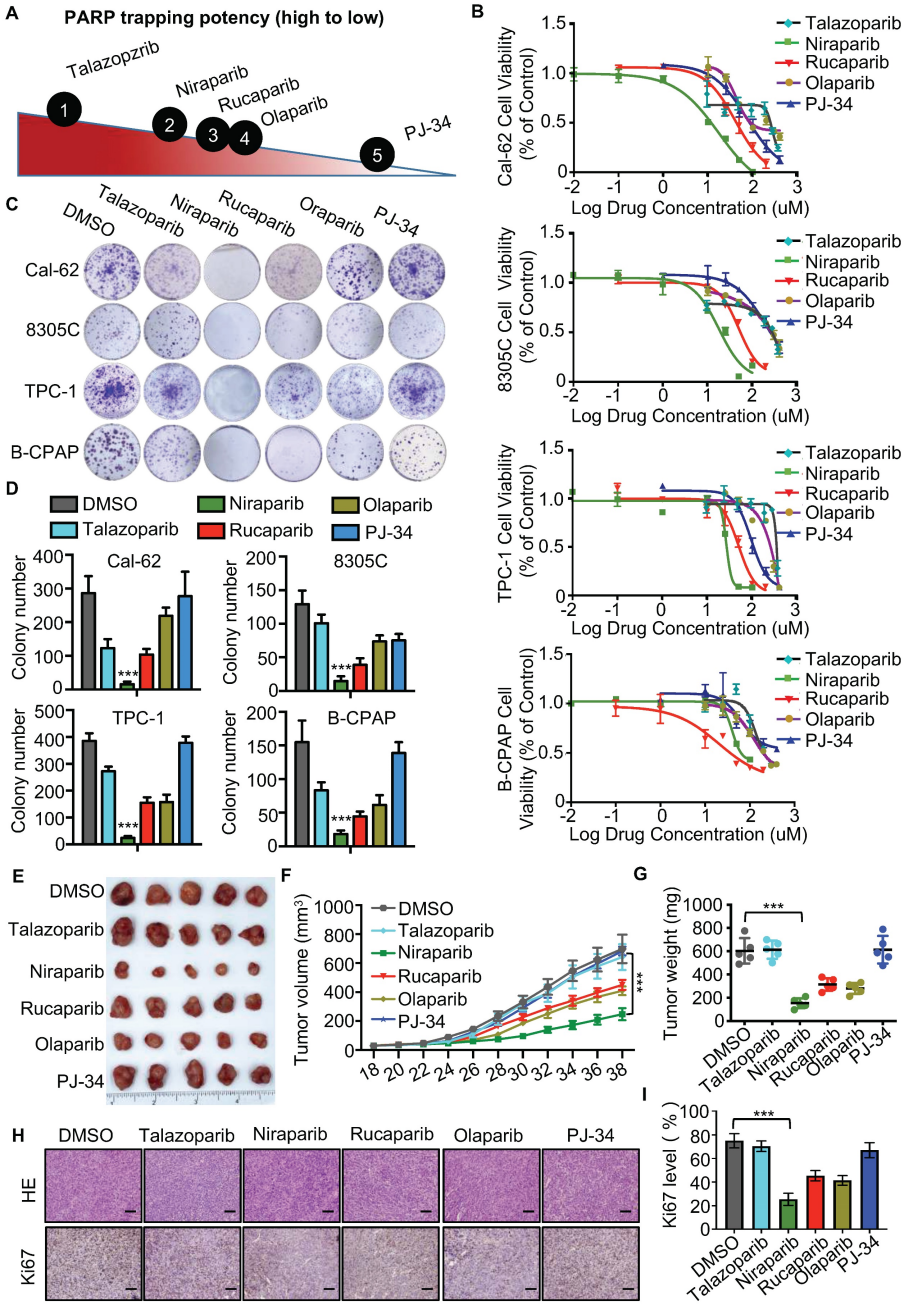
** PARP inhibitors block the growth of thyroid cancer cells *in vitro* and *in vivo*.** (A) The ability of each PARP inhibitor to trap PARP1 on DNA. (B) Comparison of IC50 of PARP inhibitors in thyroid cancer cell lines. Cell lines were exposed to PARP inhibitors for 48 h to access the IC50 values. All values are averages of replicates relative to cell viability values in DMSO treated cells normalized to 100%. (C) Representative images of colony formation of thyroid cancer cells treated with PARP inhibitors. Cell lines were exposed to PARP inhibitors with 10 uM. (D) Quantification of colony numbers of thyroid cancer cells treated with PARP inhibitors. (E) Representative tumors from mice injected with Cal-62 cells and treated with PARP inhibitors. Each group included five mice. Tumor growth curves (F) and tumor weights (G) of PARP inhibitors treatment. (H) Representative images of HE staining and IHC of Ki67 of tumors treated with PARP inhibitors. (I) Quantification of Ki67 expression level of tumor specimens treated with PARP inhibitors. The data are presented as the mean ± SD. All *p < 0.05, **p < 0.01, ***p < 0.001.

The different PARP inhibitors currently available in the clinic effectively inhibit the enzyme activity of PARP but vary in their ability to trap PARP on DNA (Fig. 1A). Cell viability assay indicated that niraparib is the most potent PARP inhibitor in thyroid cancer according to the IC50 values (Fig. 1B). And also, the colony formation assay revealed that niraparib, rucaparib, and olaparib significantly inhibit the proliferation of thyroid cancer and niraparib is the most effective (Fig. 1C and D). Furtherly, the xenograft tumor model in mice was conducted to verify the effect of PARP inhibitors on thyroid cancer. Compared with those of the DMSO treated group, the volumes and weights of niraparib, rucaparib, and olaparib treated tumors were significantly decreased (Fig. 1E-G). In addition, xenografts isolated from niraparib, rucaparib, and olaparib treated mice showed significantly fewer proliferating cells, as measured by Ki-67 with IHC staining (Fig. 1H-I). Based on niraparib is the most effective both* in vitro* and *in vivo*, therefore, in this research, we focused specially on niraparib to detect the effect and mechanism of PARPi in thyroid cancer.

### Niraparib promotes the accumulation of DNA damage in thyroid cancer cells *in vitro* and *in vivo*

**Figure 2:**
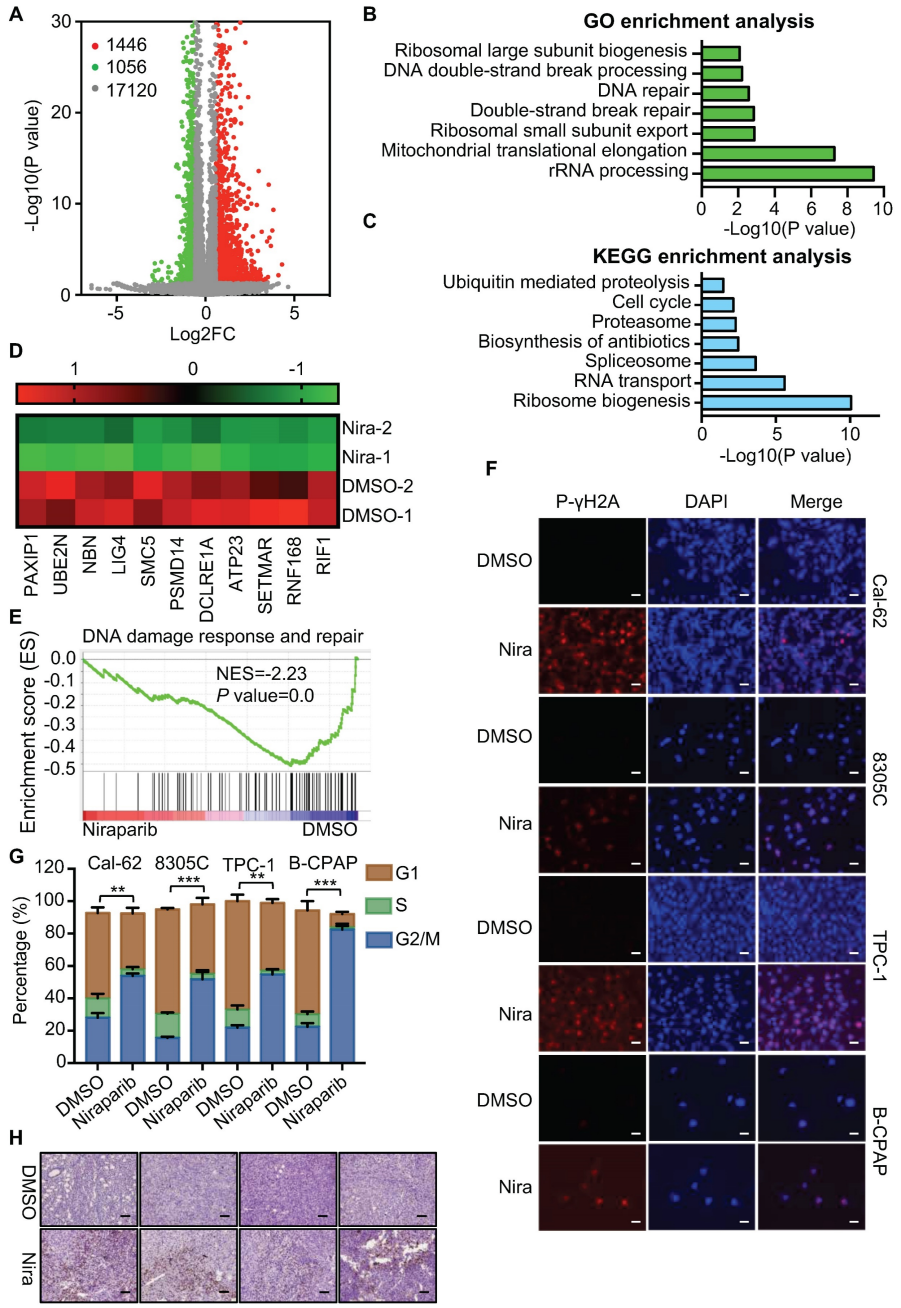
** Niraparib promotes the accumulation of DNA damage in thyroid cancer cells *in vitro* and *in vivo*.** (A) Volcano plot of the gene expression changes that occurred in Cal-62 cells treated with niraparib assayed by RNA-Seq. Summary of the GO enrichment analysis (B) and KEGG pathway enrichment analysis (C) of differentially expressed genes in Cal-62 cells treated with niraparib. (D) Heatmap from the RNA-Seq data showing the differentially expressed genes involved in the double-strand break repair process. (E) GSEA determined significant enrichment for DNA damage response and repair genes correlated with niraparib treatment. (F) IF assay determined phospho-γH2A level of thyroid cancer cell lines treated with niraparib. Cell lines were exposed to PARP inhibitors with 5 uM for 48h. (G) Analysis of cell cycle distribution of thyroid cancer cell lines treated with niraparib by flow cytometry. Cell lines were exposed to PARP inhibitors with 5 uM for 48h. (H) Representative images of IHC of phospho-γH2A of tumors treated with niraparib. The data are presented as the mean ± SD. All *p < 0.05, **p < 0.01, ***p < 0.001.

The effective concentration of niraparib is 5 uM Supplementary Fig. 1A and B). To elucidate the function and mechanism of niraparib in thyroid cancer, we performed transcriptome analysis through high-throughput RNA-Seq in Cal-62 cells treated with niraparib. Transcriptome analysis revealed that 1446 genes were upregulated, while 1056 genes were downregulated (Fig. 2A). GO enrichment analysis indicated that double-strand break repair, DNA repair, and DNA double-strand break processing were significantly enriched in downregulated genes (Fig. 2B). Further analysis revealed that the cell cycle which is coordinated with DNA damage repair progression was also significantly enriched in downregulated genes (Fig. 2C). Representative double-strand break repair genes were shown in Fig. 2D and qPCR was conducted to confirm the differential genes (Supplementary Fig. 2A). Meanwhile, GSEA showed that niraparib treatment was correlated with DNA damage response and repair (Fig. 2E).

To verify these findings of transcriptome analysis, IF was conducted and showed that DNA damage was accumulated in thyroid cancer cells upon niraparib treatment (Fig. 2F). Western blot showed that niraparib significantly increased phosphor-γH2A level (Supplementary Fig. 2B). Furtherly, comet assay revealed that niraparib significantly increased DNA damage level in thyroid cancer (Supplementary Fig. 2C). And also, flow cytometry revealed that niraparib leads to G2/M stage arrest of thyroid cancer cells (Fig. 2G). Furthermore, xenografts isolated from niraparib treated mice showed a significant accumulation of DNA damage, as measured by phospho-γH2A with IHC staining (Fig. 2H).

### Niraparib shuts down the global translation of thyroid cancer through DIMT1

**Figure 3:**
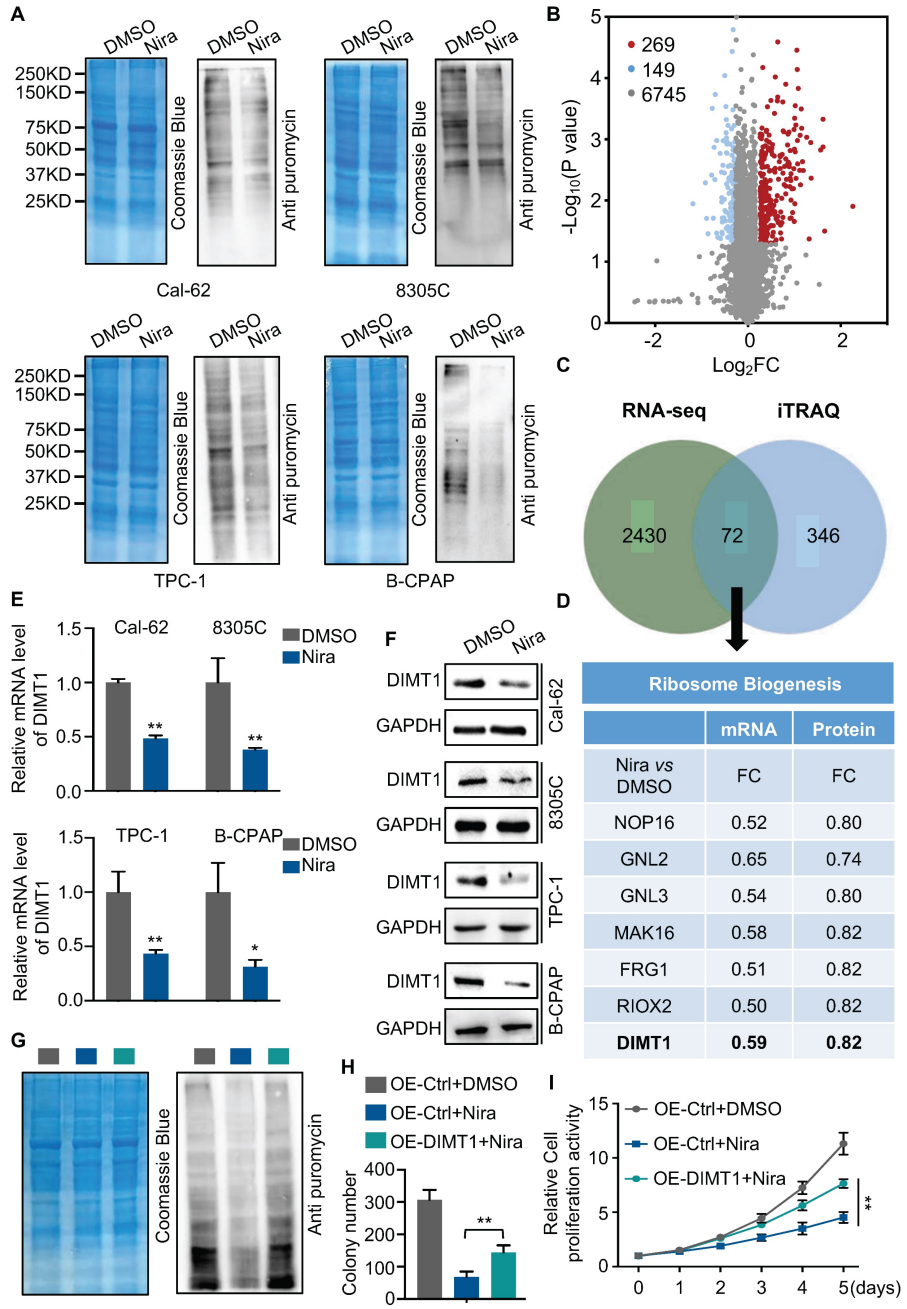
** Niraparib shuts down the global translation of thyroid cancer through DIMT1.** (A) WB-SUnSET assay in thyroid cancer cells treated with DMSO and niraparib. (B) Proteins are ranked in a volcano plot according to their statistical P-value (y-axis) and their relative abundance ratio (log_2_ fold change) between niraparib and DMSO phenotypes (x-axis). (C) Venn diagrams indicate the overlap in differentially expressed genes of transcriptome and proteome of Cal-62 cells treated with DMSO and niraparib. (D) Ribosome biogenesis-relative genes are down-regulated in both transcriptome and proteome. (E) Quantitative real-time PCR of DIMT1 in thyroid cancer cells treated with DMSO and niraparib. (F) Western blot of DIMT1 in thyroid cancer cells treated with DMSO and niraparib. (G) WB-SUnSET assay indicated that the over-expression of DIMT1 partially reversed the inhibited translation level of niraparib in Cal-62 cells. The gray bar represents Cal-62 cells expressing vector and treated with DMSO. The blue bar represents Cal-62 cells expressing vector and treated with niraparib. The green bar represents Cal-62 cells expressing DIMT1 and treated with niraparib. Colony formation assay (H) and CCK-8 assay (I) indicated that the over-expression of DIMT1 partially reversed the inhibited proliferation of niraparib in Cal-62 cells. The data are presented as the mean ± SD. All *p < 0.05, **p < 0.01, ***p < 0.001.

The GO and KEGG enrichment analysis of transcriptome showed that rRNA processing, ribosomal small unit export, and ribosome biogenesis were significantly enriched in downregulated genes (Fig. 2B and C). To access the function of niraparib, we measured the protein synthesis by SUnSET (SUrface SEnsing of Translation), a technique specifically involving the use of an anti-puromycin antibody for the immunological detection of puromycin-labeled peptides. We found that niraparib decreased the global translation level of thyroid cancer cells (Fig. 3A).

To better understand the biological consequences of niraparib on protein synthesis, we performed TMT labeling followed by mass spectrometry sequencing. The TMT-MS data revealed that 269 proteins were upregulated and 149 proteins were downregulated (Fig. 3B). The overlap of niraparib regulated genes of transcriptome and proteome including 72 genes (Fig. 3C). Mountains of genes are enriched in ribosome biogenesis. Among these genes, we focused on DIMT1 which is a key gene for ribosome biogenesis (Fig. 3D). Both qPCR and western blot revealed that niraparib significantly decreased the level of DIMT1 (Fig. 3E and F). Furthermore, we investigated the function of niraparib in thyroid cancer cells mediated by DIMT1. We overexpressed DIMT1 in thyroid cancer cells (Supplementary Fig. 3A). SUnSET assay revealed that the overexpression of DIMT1 significantly reversed the inhibition of protein synthesis by niraparib (Fig. 3G and Supplementary Fig. 3B). Meanwhile, the colony formation assay and CCK-8 assay indicated that the overexpression of DIMT1 partially reversed the proliferation ability of thyroid cancer cells (Fig. 3H-I and Supplementary Fig. 3C-3D).

### Niraparib inhibits the transcription of DIMT1 through promoting Pol II pausing in a PAR-dependent manner

**Figure 4:**
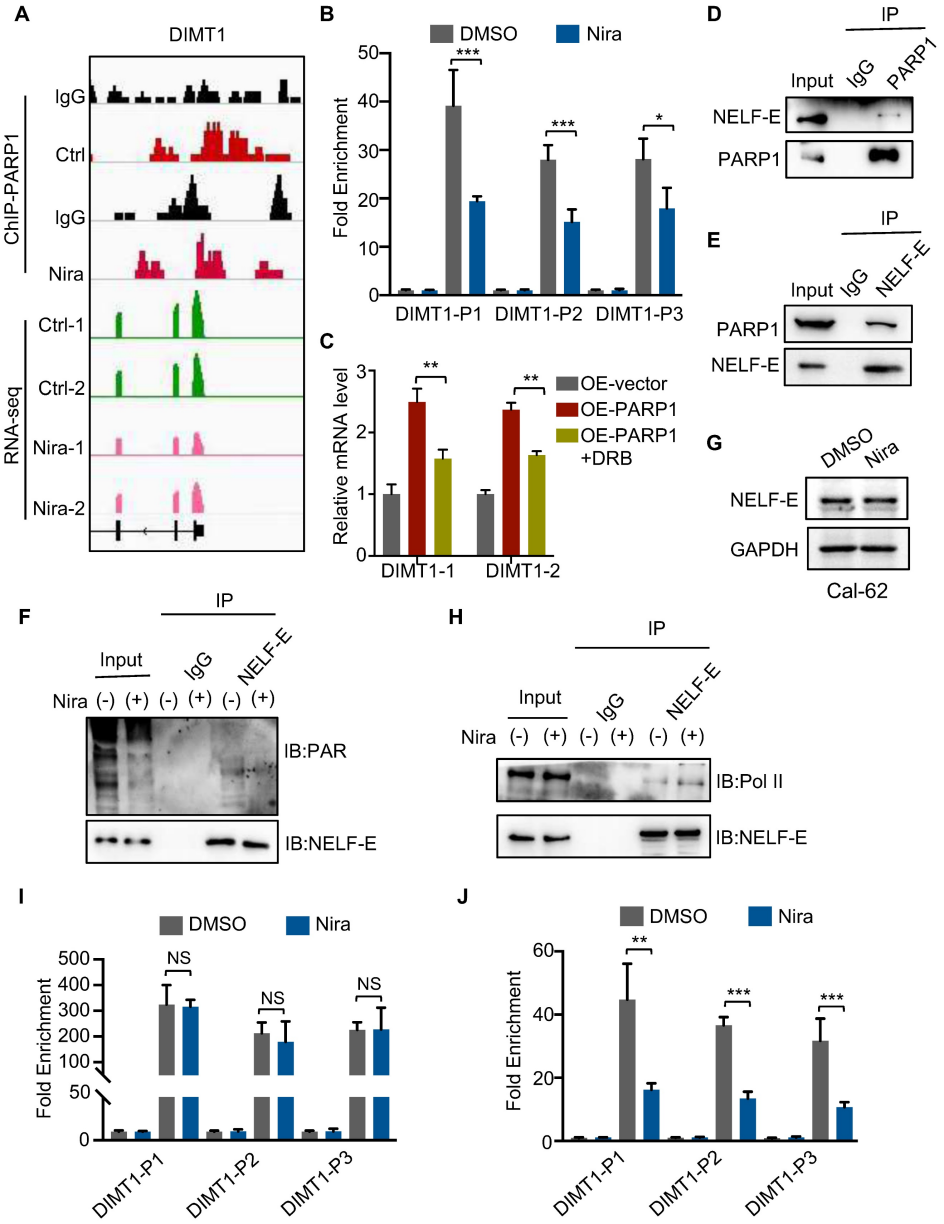
** Niraparib inhibits the transcription of DIMT1 through promoting Pol II pausing in a PAR-dependent manner.** (A) Genome browser tracks of PARP-1 ChIP-seq data and RNA-seq data around the DIMT1 gene in Cal-62 cells treated with DMSO and niraparib are shown. (B) ChIP-qPCR was performed with Cal-62 cells using PARP1 and IgG antibodies to determine the expression levels of DIMT1 in Cal-62 cells treated with DMSO and niraparib. The results were presented relative to the IgG samples. (C) The effect of DRB on the expression of DIMT1 induced by PARP1 overexpression as determined by qRT-PCR. (D-E) Cal-62 cells were lysed with RIPA buffer, and lysates were subjected to immunoprecipitation using either anti-IgG, anti-PARP1, or anti-NELF-E antibodies, and analyzed by western blot with indicated antibodies. (F) Cal-62 cells treated with niraparib or DMSO and lysates were subjected to immunoprecipitation using anti-NELF-E and analyzed by western blot with indicated antibodies. (G) Western blot of NELF-E in Cal-62 cells treated with DMSO and niraparib. (H) Cal-62 cells treated with niraparib or DMSO and lysates were subjected to immunoprecipitation using anti-NELF-E and analyzed by western blot with indicated antibodies. ChIP-qPCR was performed with Cal-62 cells using Pol II (I), phosphoS2 Pol II (J), and IgG antibodies to determine the expression levels of DIMT1 in Cal-62 cells treated with DMSO and niraparib. The results were presented relative to the IgG samples. The data are presented as the mean ± SD. All *p < 0.05, **p < 0.01, ***p < 0.001.

To further detect the molecular mechanism between niraparib and DIMT1, we performed a ChIP-seq of PARP1 in Cal-62 cells treated with DMSO and niraparib. Genome browser snapshots of ChIP-seq and RNA-seq showed that PARP1 is enriched in the promoter of the DIMT1 gene, and niraparib led to a marked reduction in PARP1 binding to the promoter region of the DIMT1 gene (Fig. 4A). The inhibition of the enrichment of PARP1 on DIMT1 by niraparib was furtherly verified by ChIP-qPCR (Fig. 4B). Recently, research revealed that depletion or inhibition of PARP-1 promotes Pol II pausing (26. Here, we found that the inhibitor of Pol II-DRB significantly reversed the upregulation of DIMT1 induced by PARP1 overexpression (Fig. 4C). NELF-E is proposed to be required for the establishment of pausing by Pol II and the mutation of the ADP-ribosylation sites on NELF-E promotes Pol II pausing 26, 27. Our Co-IP data indicated that PARP1 interacted with NELF-E and mediated the PARylation of NELF-E which could be abolished by niraparib (Fig. 4D-F). However, the western blot showed that niraparib does not affect the NELF-E level (Fig. 4G). Meanwhile, the Co-IP assay also showed that NELF-E interacted with Pol II and niraparib enhanced the interaction between NELF-E and Pol II (Fig. 4H). Moreover, our ChIP-qPCR data revealed that niraparib has no effect on the occupancy of Pol II on DIMT1, while the occupancy of phosphoS2 Pol II, standing for the activated Pol II, on DIMT1 was significantly decreased (Fig. 4I-J). In summary, we proved that niraparib inhibits the PARylation of NELF-E which enhanced the interaction between NELF-E and Pol II, subsequently promoting Pol II binding to DIMT1 pause.

### PARP1 regulates p65 protein stability through the ubiquitination-dependent pathway

**Figure 5:**
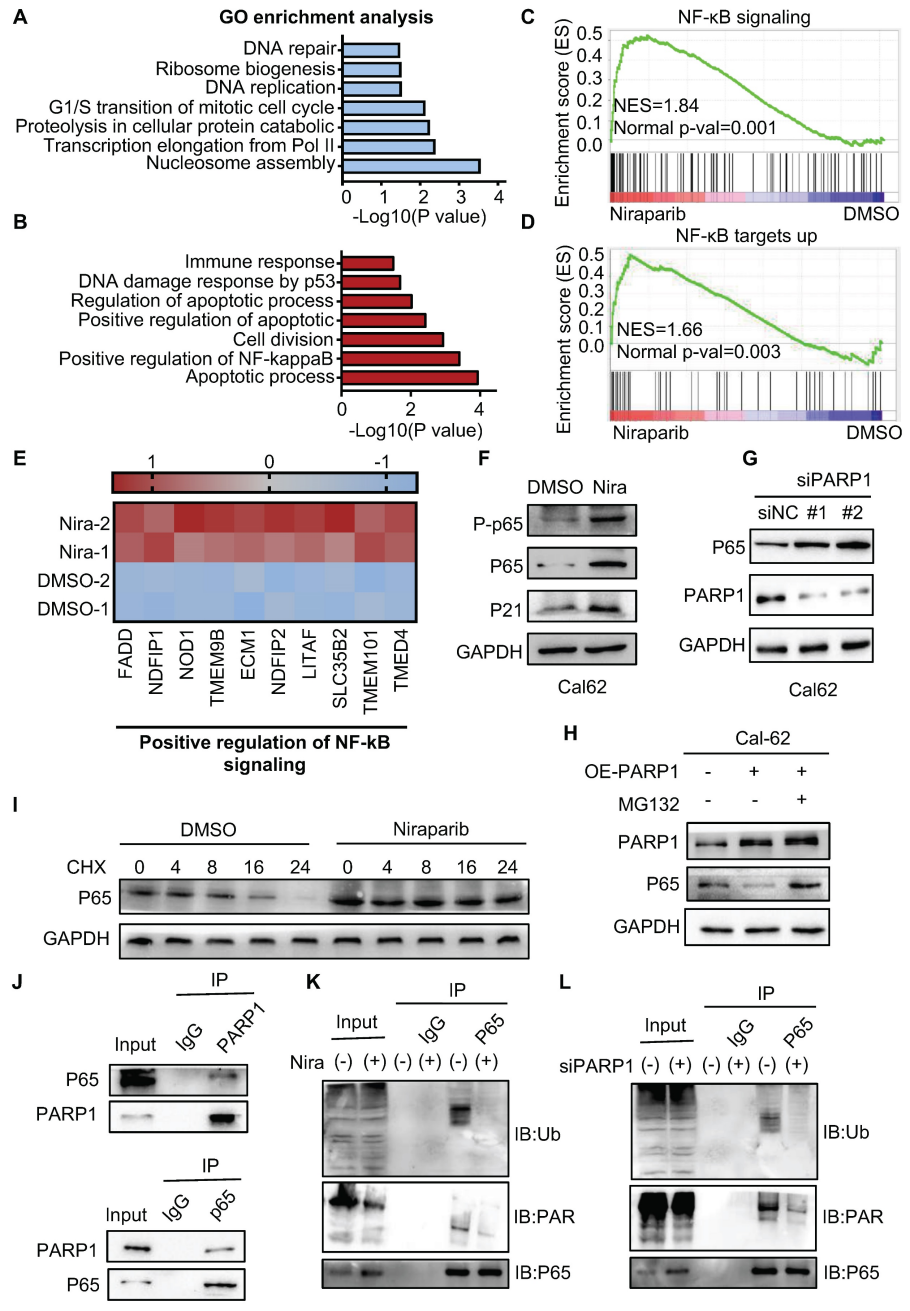
** PARP1 regulates p65 protein stability through the ubiquitination-dependent pathway.** Summary of the proteomic GO enrichment analysis of downregulated genes (A) and upregulated genes (B) in Cal-62 cells treated with niraparib. (C) GSEA determined significant enrichment for NF-κB signaling genes correlated with niraparib treatment. (D) GSEA determined significant enrichment for NF-κB targets up genes correlated with niraparib treatment. (E) Heatmap from the proteome data showing the differentially expressed genes involved in the positive regulation of NF-κB signaling. (F) Western blot of phosphorylated p65, p65, and p21 of Cal-62 cells treated with niraparib. (G) Cal-62 cells were transfected with either scrambled or PARP1 siRNAs for 48 h, and protein levels were detected by western blot with the indicated antibodies. (H) Cal-62 cells overexpressing flag-PARP1 were treated with MG132 (10 uM) for 4 h, and proteins were detected by western blot with indicated antibodies. (I) Cal-62 cells treated with niraparib or DMSO were incubated with 10 ug/ml cycloheximide (CHX) for the indicated times. Lysates were harvested and analyzed by western blot. (J) Cal-62 cells were lysed with RIPA buffer, and lysates were subjected to immunoprecipitation using either anti-IgG, anti-PARP1, or anti-p65 antibodies, and analyzed by western blot with indicated antibodies. (K) Cal-62 cells treated with niraparib or DMSO and lysates were subjected to immunoprecipitation using anti-p65 and analyzed by western blot with indicated antibodies. (L) Cal-62 cells were transfected with either scrambled or PARP1 siRNA for 48 h, and lysates were subjected to immunoprecipitation using anti-p65 and analyzed by western blot with indicated antibodies. The data are presented as the mean ± SD. All *p < 0.05, **p < 0.01, ***p < 0.001.

To better understand the mechanism of niraparib in thyroid cancer, we performed proteome analysis. GO enrichment analysis showed that cell cycle, ribosome biogenesis, and DNA repair were significantly enriched which is consistent with what we have found in the transcriptome (Fig. 5A).

Apart from that, positive regulation of NF-κB signaling was significantly enriched in the upregulated genes (Fig. 5B). Meanwhile, GSEA also showed that NF-κB signaling and NF-κB targets up were significantly enriched (Fig. 5C-D). Further analysis revealed that niraparib significantly increased NF-κB signaling pathway gene expression and NF-κB target gene expression (Fig. 5E and Supplementary Fig. 4A). Indeed, western blot showed that niraparib activated the NF-κB signaling which was identified by the upregulation of phosphorylated p65, p65, and p21 (Fig. 5F and Supplementary Fig. 4B). Moreover, knockdown PARP1 with siRNA has also increased the level of p65 (Fig. 5G). This negative regulation of p65 by PARP1 did not occur at the transcriptional level, as the mRNA level of p65 was not altered with niraparib which was revealed by RAN-seq Supplementary Fig. 4C).

PARylation as a crucial post-translational modification, has been reported to regulate the transcriptional activity, cellular localization, and protein stabilization. Therefore, we tested whether PARP1 contributed to p65 degradation. As shown in Fig. 5H, overexpression of PARP1 markedly declined the level of the p65 protein. MG132, a specific proteasome inhibitor, substantially rescued the decline of the p65 protein caused by overexpression of PARP1 in Cal-62 cells. Consistent with this notion, the half-life of endogenous p65 protein became longer in niraparib treated or PARP1 knockdown cells in the presence of cycloheximide, an inhibitor of protein synthesis (Fig. 5I and Supplementary Fig. 4D).

Next, we examined the interaction between PARP1 and p65, a complex containing p65 and PARP1 was clearly detected in HEK293T cells expressing flag-tagged PARP1 (Supplementary Fig. 4E). Consistently, both endogenous PARP1 and p65 were clearly detected in the immunoprecipitated complex in Cal-62 cells (Fig. 5J). Ubiquitination-mediated proteasomal degradation of proteins is a common mechanism that regulates protein stability. Studies showed that PARylated proteins lead to their degradation via a proteasome-dependent pathway (28-30. To further determine whether the PARylation of p65 lead to the ubiquitination-mediated degradation itself, we conducted the co-IP experiment and found that PARylation-mediated degradation of p65 occurs through poly-ubiquitination (Fig. 5K and L).

### E3 ligase RNF146 promotes p65 ubiquitination and degradation

**Figure 6:**
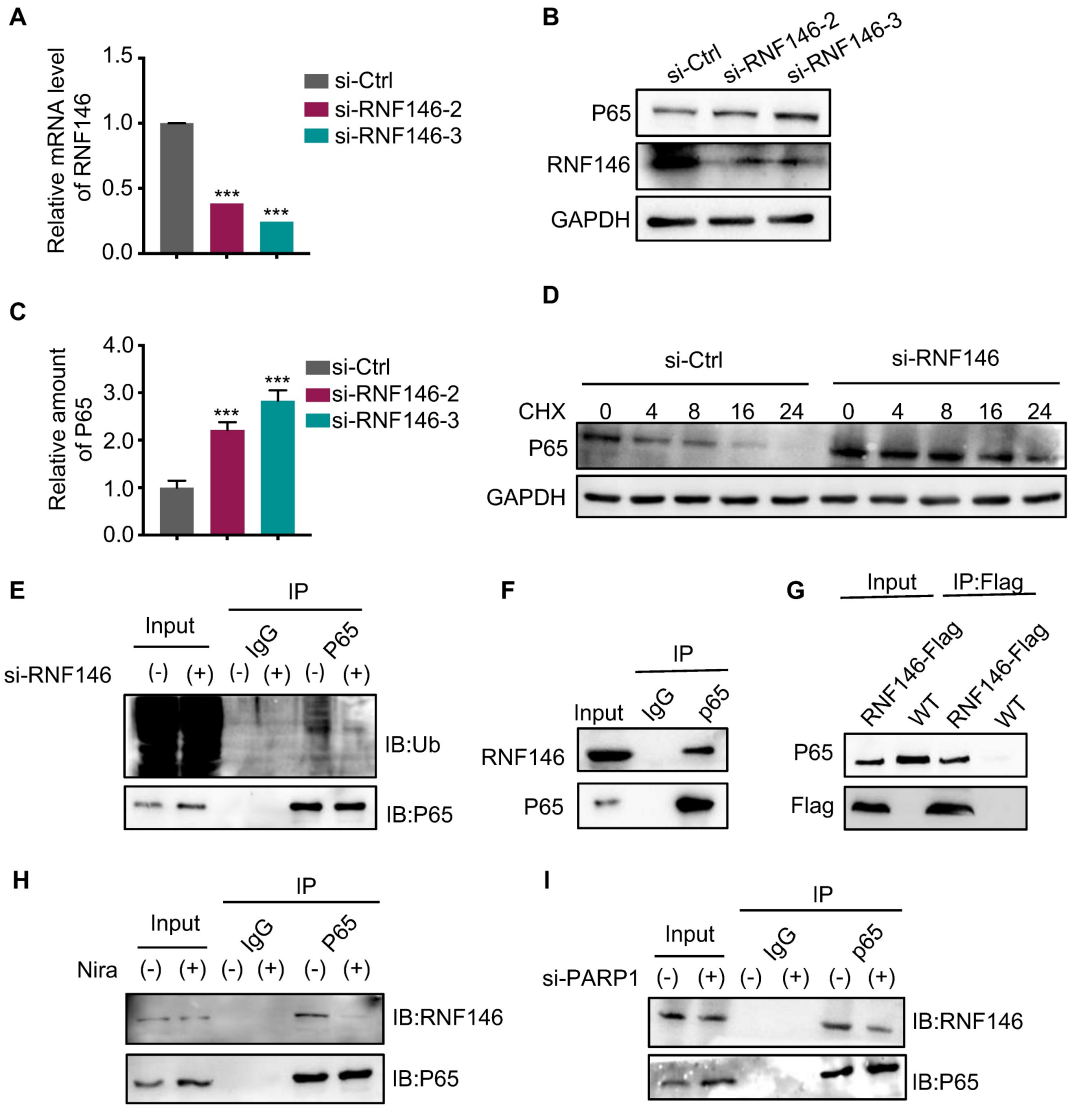
** E3 ligase RNF146 promotes p65 ubiquitination and degradation.** (A) Quantitative real-time PCR of RNF146 in Cal-62 cells transfected with either scrambled or RNF146 siRNA for 48 h. (B) Cal-62 cells were transfected with either scrambled or RNF146 siRNAs (48 h). Protein levels were detected by western blot with indicated antibodies. (C) Quantification of p65 protein levels from Fig (B). Relative amounts normalized to the p65 protein level of the control group. (D) Cal-62 cells were transfected with either scrambled or RNF146 siRNAs for 48 h, followed by incubation with 10 ug/ml cycloheximide (CHX) for the indicated periods of time. Lysates were harvested and analyzed by western blot. (E) Cal-62 cells transfected with either RNF146 or control siRNA for 24 h, MG132 (10 uM) was added for 4 h and lysed with RIPA, followed by anti-p65 IP and analyzed by western blot with the indicated antibodies. (F) Cal-62 cells were lysed with RIPA buffer, and lysates were subjected to immunoprecipitation using either anti-IgG, or p65 antibodies, and analyzed by western blot with indicated antibodies. (G) Cal-62 cells expressing RNF146-flag were lysed with RIPA buffer, and lysates were subjected to immunoprecipitation using flag antibody, and analyzed by western blot with indicated antibodies. (H) Cal-62 cells treated with niraparib or DMSO and lysates were subjected to immunoprecipitation using anti-p65 and analyzed by western blot with indicated antibodies. (I) Cal-62 cells were transfected with either scrambled or PARP1 siRNA for 48 h, and lysates were subjected to immunoprecipitation using anti-p65 and analyzed by western blot with indicated antibodies. The data are presented as the mean ± SD. All *p < 0.05, **p < 0.01, ***p < 0.001.

An increasing number of studies revealed that the E3 ligase RNF146 functions by interacting with PARylated proteins and promoting their degradation 28, 29, 31. Therefore, we examined whether RNF146 can act as an E3 ligase of p65. We found that the silence of RNF146 led to p65 stabilization (Fig. 6A, B and C). Meanwhile, the half-life of endogenous p65 protein increased after depleting RNF146 in Cal-62 cells in the presence of cycloheximide (Fig. 6D). Moreover, the silence of RNF146 significantly decreased the ubiquitination level of p65 (Fig. 6E). The co-IP assay revealed that p65 interacted with RNF146 (Fig. 6F-G). And niraparib treatment or PARP1 knockdown markedly inhibited the interaction between p65 and RNF146 (Fig. 6H and I). Here, we elucidated that RNF146 promotes p65 ubiquitination and degradation depending on PARP1-mediated ribosylation of p65.

### Bortezomib enhances the antitumor effect of niraparib* in vitro* and *in vivo*

**Figure 7:**
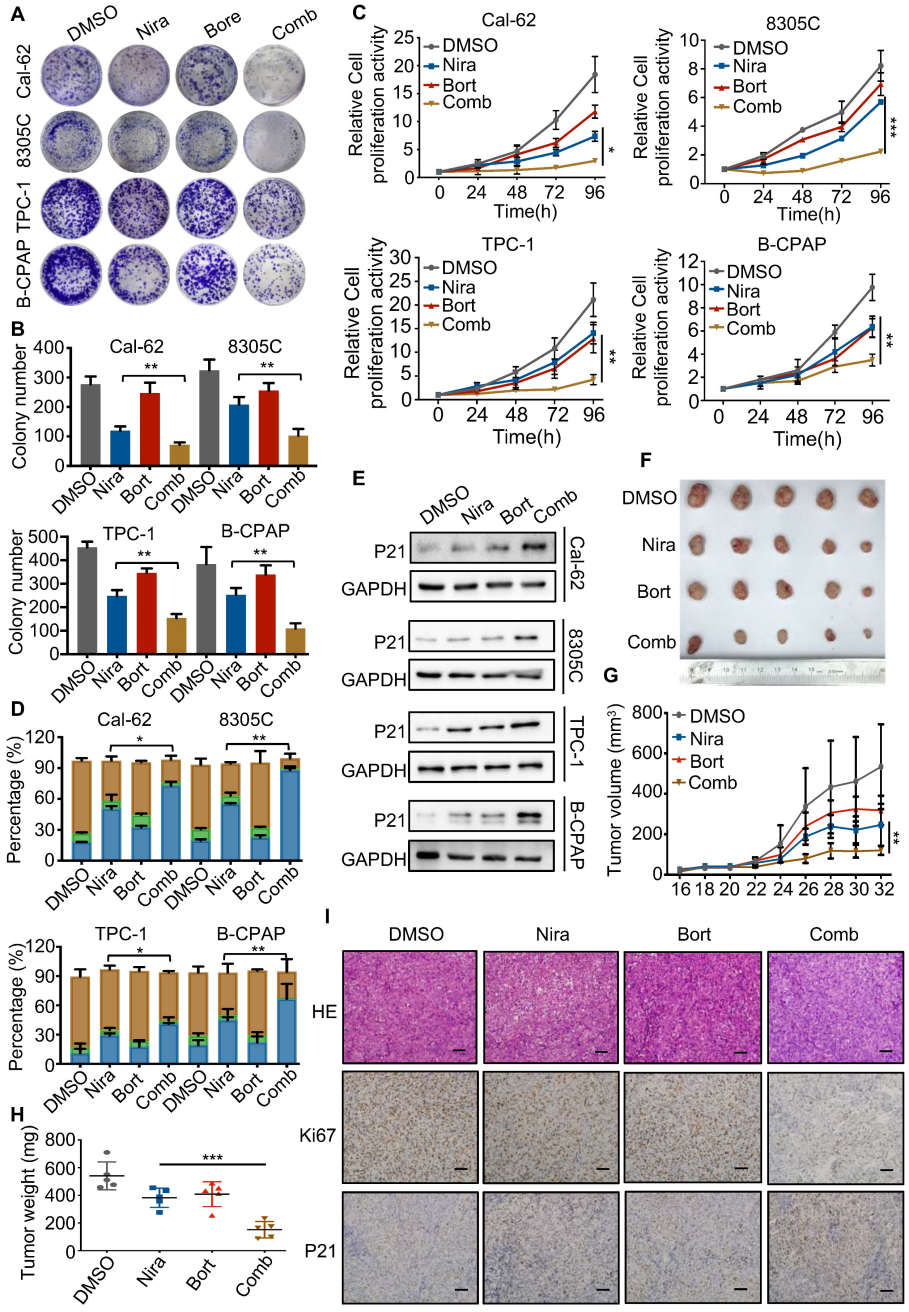
** Bortezomib enhances the antitumor effect of niraparib* in vitro* and *in vivo*.** (A) Representative images of colony formation of thyroid cancer cells treated with niraparib and bortezomib. (B) Quantification of colony numbers of thyroid cancer cells treated with niraparib and bortezomib. (C) CCK-8 assays determined the proliferation of thyroid cancer cells treated with niraparib and bortezomib. (D) Analysis of cell cycle distribution of thyroid cancer cell lines treated with niraparib and bortezomib. (E) Western blot was used to determine the p21 level of thyroid cancer cell lines treated with niraparib and bortezomib. (F) Representative tumors from mice injected with Cal-62 cells and treated with niraparib and bortezomib. Each group included five mice. Tumor growth curves (G) and tumor weights (H) of niraparib and bortezomib treatment. (I) Representative images of HE staining and IHC of Ki67 and p21 of tumors treated with niraparib and bortezomib. The data are presented as the mean ± SD. All *p < 0.05, **p < 0.01, ***p < 0.001.

The activation of the NF-κB pathway has been reported in response to a variety of anti-cancer therapeutic agents and its inhibition has been observed to re-sensitize drug-resistant cells to anti-cancer agents. Therefore, blocking the NF-κB signaling pathway may help sensitize thyroid cancer cells to PARP inhibitors. The colony formation assay indicated that bortezomib, a NF-κB pathway inhibitor, dramatically enhanced the growth inhibition of niraparib in TCs (Fig. 7A and B). Meanwhile, the CCK-8 assay also showed that bortezomib significantly enhanced the growth inhibition of niraparib (Fig. 7C). Next, we used flow cytometry to detect the cell cycle distribution and found that bortezomib and niraparib alone could induce G2/M stage arrest to some extent, while their combination led to a better response (Fig. 7D). Western blot analysis also indicated that bortezomib combined with niraparib markedly increased the p21 level (Fig. 7E). Moreover, to assess whether the effect of the combination of bortezomib and niraparib *in vivo* is synergistic, 20 female BALB/c nude mice were subcutaneously injected with Cal-62 cells and treated with bortezomib and niraparib (Fig. 7F). The results revealed that the tumor volumes and tumor weights were significantly inhibited by niraparib, while the combination of bortezomib and niraparib resulted in greater tumor suppression (Fig. 7G and H). IHC staining of xenografts indicated that the combination of bortezomib and niraparib could significantly decrease the expression of Ki-67 and increase the expression level of p21 *in vivo* (Fig. 7I). Overall, bortezomib could significantly enhance the antitumor effect of niraparib *in vitro* and *in vivo*.

## Discussion

**Figure 8:**
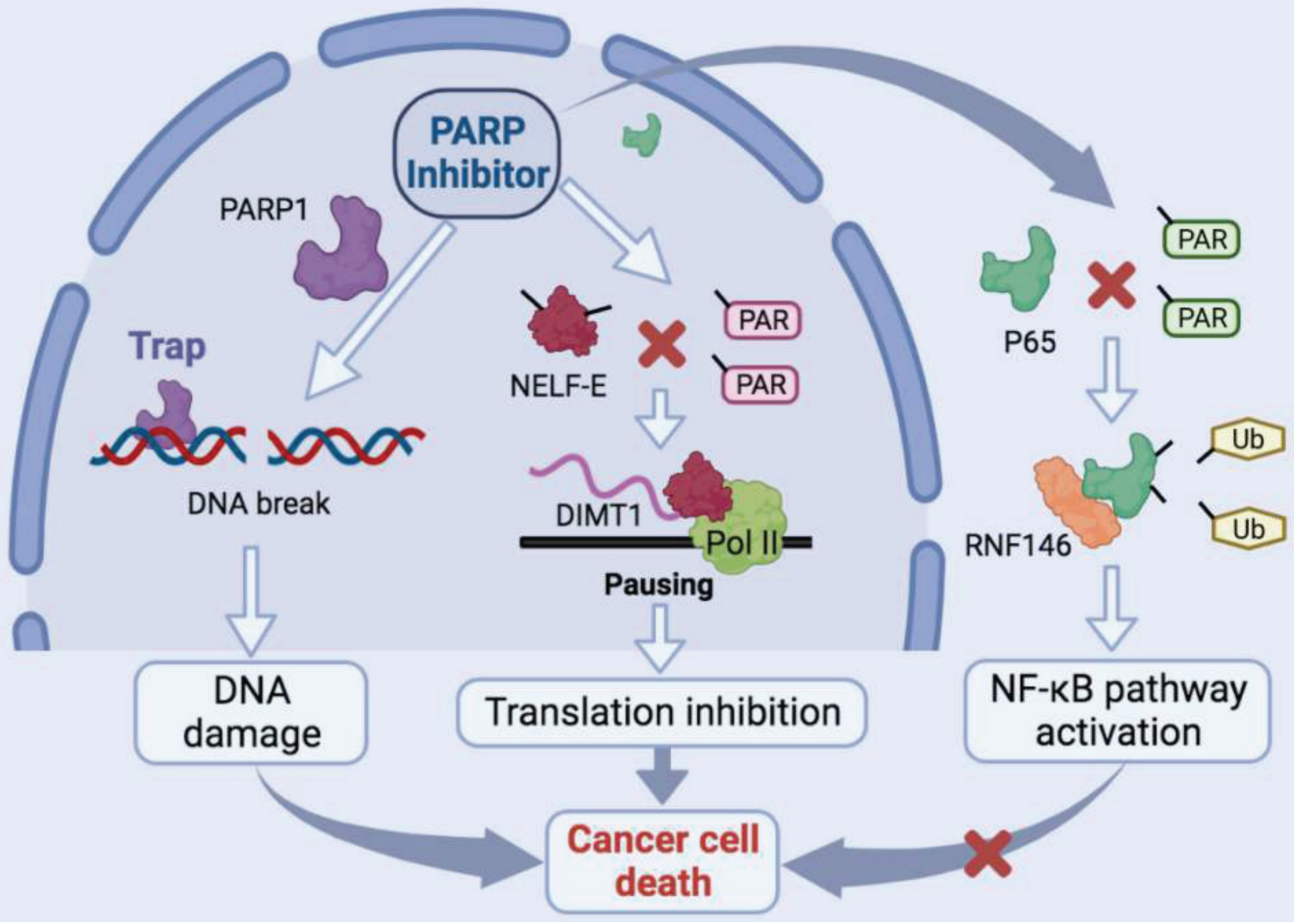
** Schematic model of the function and mechanism of PARP inhibitor in thyroid cancers.** Niraparib not only promotes the DNA damage accumulation in TCs but also inhibits the transcription of DIMT1 through promoting Pol II pausing in a PAR-dependent manner, which leads to a global translation shut-down in TCs. Meanwhile, niraparib activates the NF-κB signaling pathway by inhibiting the PARylation of p65, which decreases its ubiquitination and degradation level through E3 ubiquitin ligase RNF146. Moreover, inhibition of the NF-κB pathway augmented the antitumor effect of niraparib in TCs.

In this study, we revealed that PARP inhibitors own great therapeutic value for TCs. Niraparib not only promotes the DNA damage accumulation in TCs but also inhibits the transcription of DIMT1 through promoting Pol II pausing in a PAR-dependent manner, which leads to a global translation shut-down in TCs. Meanwhile, we found that niraparib activates the NF-κB signaling pathway by inhibiting the PARylation of p65, which decreases its ubiquitination and degradation level through E3 ubiquitin ligase RNF146. Moreover, inhibition of the NF-κB pathway augmented the antitumor effect of niraparib in TCs (Fig. 8).

The concept of synthetic lethal (SL) is described as the situation whereby a defect in either one of two genes is non-lethal, but a combination of defects in both genes results in death 32. The SL interaction between PARP inhibition and BRCA1 or BRCA2 mutation has been described decades ago 5, 6. Up to now, several PARP inhibitors have been applied in the clinic in various of BRCA mutant cancers and showed markedly antitumor effect 33-35. The understanding of the biological functions of BRCA genes has made considerable progress. Studies revealed that any mutations of genes leading to homologous recombination repair defect displaying BRCAness 36, 37. Therefore, any cancer type owning BRCAness genes mutation got the possibility to be sensitive to PARP inhibitors. Research revealed the mutational landscape of TCs and found that a reasonable quantity of BRCAness genes mutated in TCs which throws light on the application of PARP inhibitors in TCs 12. Our data also indicated that PARP inhibitors block the growth of TCs *in vitro* and *in vivo* partially by promoting the accumulation of DNA damage. Although the ability of talazoparib to trap PARP1 is 100 times potent than niraparib, our data showed that niraparib is the most potent in TCs. That may be due to comparing to talazoparib, niraparib was more selective for PARP1 and PARP2 which are key regulators in TCs 38. Moreover, studies revealed that different PARPi have distinct interaction profiles, and whether differences in the “off-target” profile contribute to different antitumor effect of PARPi need to be further elucidated 38, 39.

Recently, research showed that PARP inhibitors inhibit the BRCA1/2-intact breast cancer cell growth through ribosome biogenesis and rDNA transcription 16. This provides a mechanistic rationale for the therapeutic effect of PARP inhibitors in cancer cells lacking BRCA gene mutation. Therefore, we treated thyroid cancer cells with niraparib and found that niraparib significantly decreased the global translation in TCs. To detect the molecular mechanism, we performed an integrated analysis of transcriptome and proteome and found that niraparib decreased DIMT1 at both transcription and translation levels. Various researches revealed that PARP1 participates in the regulation of transcriptional processes, including as a transcription factor itself, regulation of various transcription factors, and general chromatin remodeling 40-42. Meanwhile, our ChIP-seq data of PARP1 determined niraparib led to a marked reduction in PARP1 binding to the promoter region of the DIMT1 gene. Recently, a study showed that depletion or inhibition of PARP-1 or mutation of the ADP-ribosylation sites on NELF-E promotes Pol II pausing 26. Here, we revealed that niraparib inhibits the PARylation of NELF-E which enhanced the interaction between NELF-E and Pol II, subsequently promoting Pol II binding to DIMT1 paused and shutting down the global translation of TCs. However, the ADP-ribosylation sites of NELF-E mediated Pol II pausing in TCs need to be further investigated.

Direct interactions between PARP1 and p65 have been found in several researches 43-45. Consistent with that, our data indicated that PARP1 binds and poly(ADP)ribosylates p65 in TCs. As one of the most important post-translational modifications, PARylation regulates protein stabilization, protein-protein interaction, cell fate determination, and cellular localization 46-49. The role of PARylation in proteasomal regulation is well established, PARylation not only regulates overall proteasome activity but also promotes ubiquitination and targeting of specific proteins to proteasomal degradation 31, 50. In this study, we demonstrated that PARylated p65 was recognized by the PAR-binding E3 ligase RNF146 which poly-ubiquitinated p65 leading to its degradation. Moreover, targeting PARP1 with niraparib abolished the PARylation of p65 which leads to the stabilization of p65, subsequently activating the NF-κB signaling pathway. Abnormal activation of the NF-κB signaling pathway leads to low sensitivity or even drug resistance in various cancers 51-54. Here, we revealed that bortezomib significantly enhanced the antitumor effect of niraparib in TCs *in vitro* and *in vivo*.

## Conclusions

In summary, our study revealed that PARP inhibitors own great therapeutic value for TCs. Inhibition of PARP1 significantly promotes the accumulation of DNA damage in TCs. Meanwhile, our finding revealed a novel mechanism that PARP inhibitor suppresses the PARylation of NELF-E which enhanced the interaction between NELF-E and Pol II, subsequently promoting Pol II binding to DIMT1 pause and shutting down the global translation of TCs. This provides a mechanistic rationale for the therapeutic effect of PARP inhibitors in cancer cells lacking BRCA gene mutation. Moreover, we uncovered that bortezomib combined with niraparib augmented the antitumor effect in TCs, which shed light on the potential combination strategy of PARPi in the treatment of thyroid cancers.

## Supplementary Material

Supplementary figures and tables.

## Abbreviations

TCs: Thyroid cancers
PARylation: Poly-ADP-ribosylation
PTC: Papillary thyroid cancer
ATC: Anaplastic thyroid cancer
PAR: poly (ADP-ribose)
HRD: Homologous recombination repair deficiency
Pol II: RNA polymerase II
IHC: Immunohistochemistry
CCK-8: Cell Counting Kit-8
DAVID: Database for annotation, visualization, and integrated discovery
LC/MS: Liquid chromatography-tandem mass spectrometry
PBS: Phosphate-buffered saline
SUnSET: SUrface SEnsing of Translation
DRB: Dichlorobenzimidazole 1-β-D-ribofuranosylbenzimidazole
