# Supplementary material for: PARP inhibitor shuts down the global translation of thyroid cancer through promoting Pol II binding to DIMT1 pause
Source: Int J Biol Sci. 2023 Jul 31;19(12):3970–86. doi: 10.7150/ijbs.81895 (PMC10411462; doi:10.7150/ijbs.81895)
Supplement: Supplementary file 1 — Supplementary figures and tables. [file ijbsv19p3970s1.pdf]

PARP inhibitor shuts down the global translation of thyroid cancer through promoting Pol II binding to DIMT1 pause

Xiukun Hou, Mengran Tian, Junya Ning, Zhongyu Wang, Fengli Guo, Wei Zhang, Linfei Hu, Songfeng Wei, Chuanxiang Hu, Xinwei Yun, Jingzhu Zhao, Qiman Dong, Xianhui Ruan, Dapeng Li, Ming Gao, and Xiangqian Zheng

Supplementary Figures

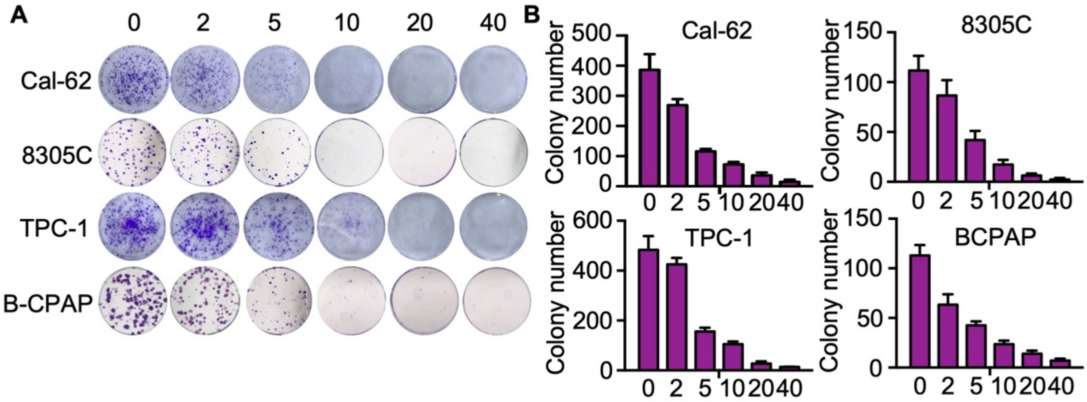

Supplementary Fig 1. PARP inhibitors block the growth of thyroid cancer cells *in vitro* and *in vivo*

(A) Colony formation assay of different concentrations of niraparib in thyroid cancer cells. (B) Quantification of colony numbers of thyroid cancer cells treated with niraparib. The data are presented as the mean ± SD. All *p < 0.05, **p < 0.01, ***p < 0.001.

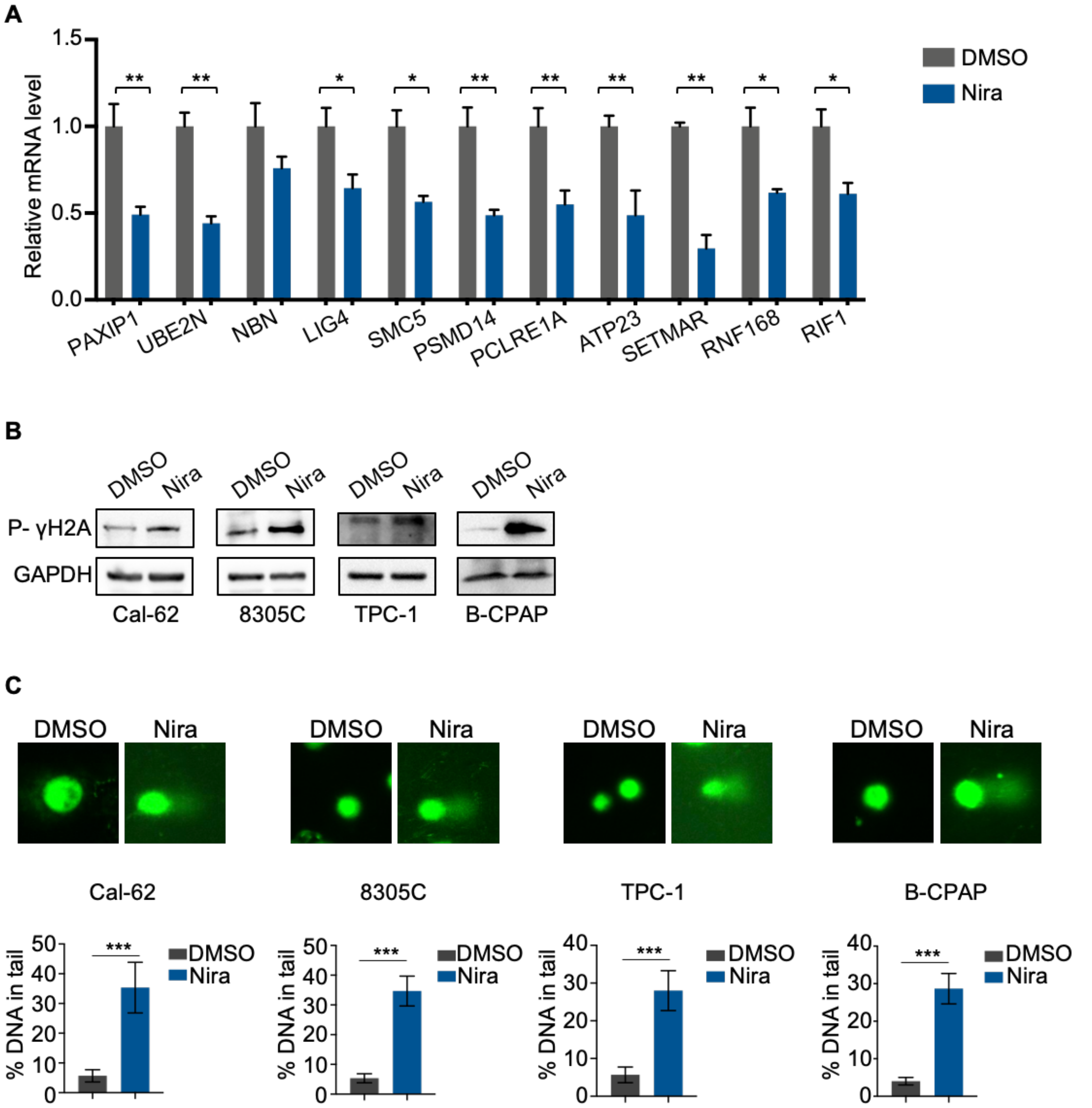

Supplementary Fig 2. Niraparib promotes the accumulation of DNA damage in thyroid cancer cells in vitro and in vivo

(A) Quantitative PCR was conducted to detect the expression level of genes involved in double-strand break repair process upon niraparib treatment; (B) Western blot determined phospho-γH2A level of thyroid cancer cell lines treated with niraparib; (C) Comet assay determined the DNA damage level of thyroid cancer cell lines treated with niraparib.

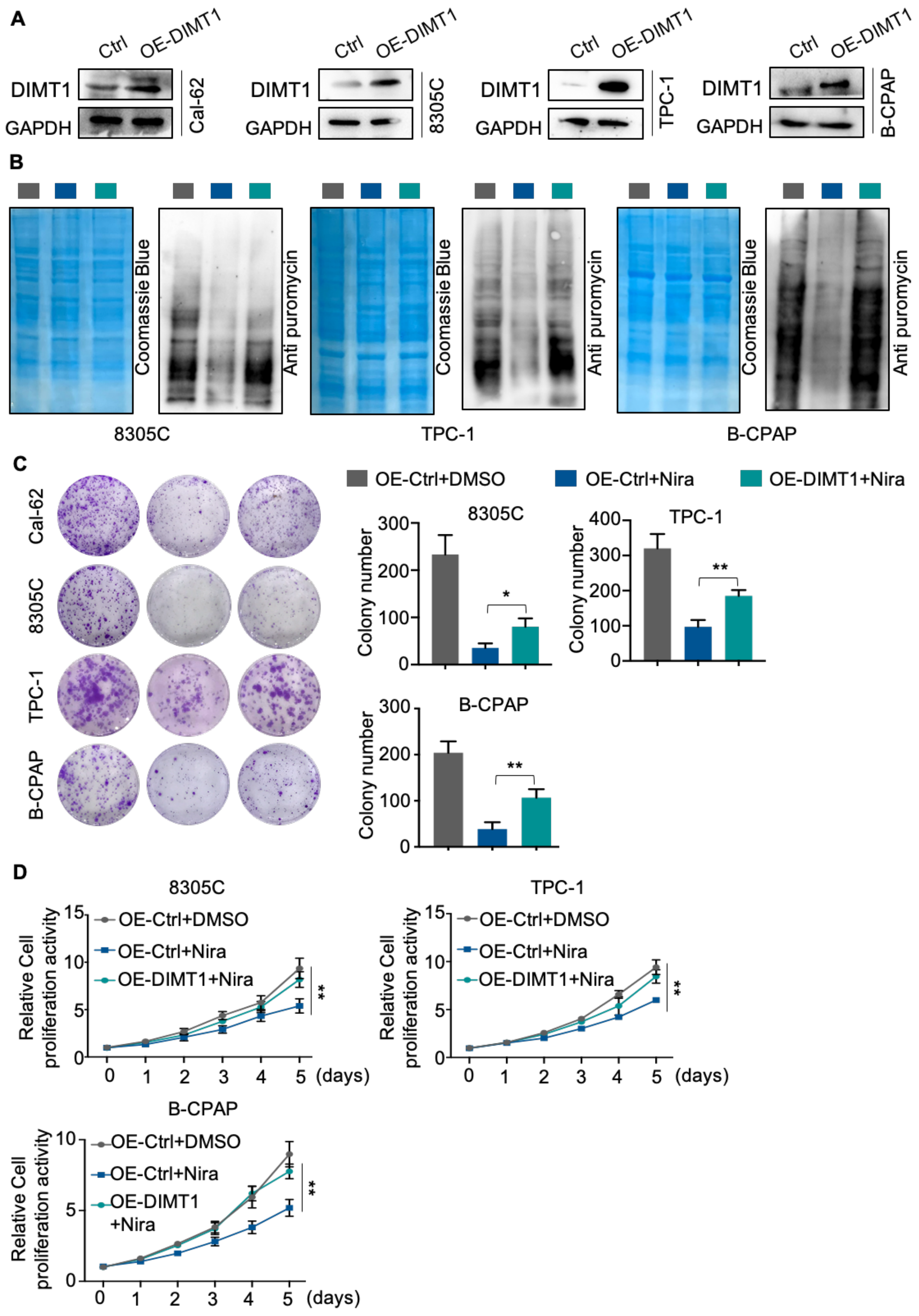

Supplementary Fig 3. Niraparib shuts down the global translation of thyroid cancer through DIMT1

(A) The western blot of DIMT1 in thyroid cancer cells overexpressing DIMT1. (B) WB-SUnSET assay indicated that the over-expression of

DIMT1 partially reversed the inhibited translation level of niraparib in 8305C cells, TPC-1 cells, and B-CPAP cells. Colony formation assay (C) and CCK-8 assay (D) indicated that the over-expression of DIMT1 partially reversed the inhibited proliferation of niraparib in 8305C cells, TPC-1 cells, and B-CPAP cells. The data are presented as the mean ± SD. All $*p < 0.05$, $**p < 0.01$, $***p < 0.001$.

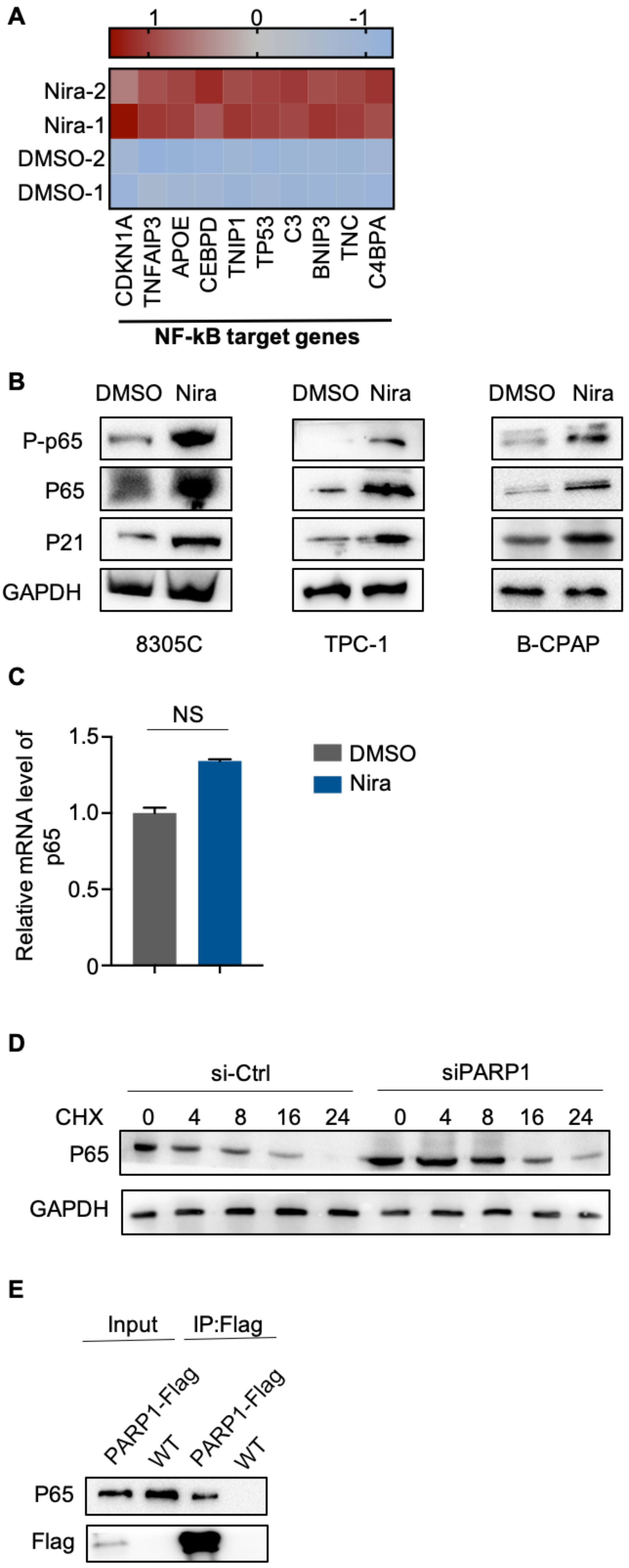
A
1
0
-1
Nira-2
Nira-1
DMSO-2
DMSO-1
CDKN1A
TNFAIP3
APOE
CEBPD
TNIP1
TP53
C3
BNIP3
TNC
C4BPA
NF-kB target genes
B
DMSO Nira
P-p65
P65
P21
GAPDH
8305C
TPC-1
B-CPAP
C
NS
Relative mRNA level of p65
1.5
1.0
0.5
0
DMSO
Nira
D
si-Ctrl
siPARP1
CHX 0 4 8 16 24 0 4 8 16 24
P65
GAPDH
E
Input
IP:Flag
PARP1-Flag
WT
PARP1-Flag
WT
P65
Flag

Supplementary Fig 4. PARP1 regulates p65 protein stability through the ubiquitination-dependent pathway.

(A) Heatmap from the proteome data showing the differentially expressed genes involved in the NF-κB target genes. (B) Western blot of phosphorylated p65, p65, and p21 of 8305C cells, TPC-1 cells, and B-CPAP cells treated with niraparib. (C) The quantification of mRNA level of p65 from RAN-seq data. (D) Cal-62 cells transfected with either scrambled or PARP1 siRNA for 48 h, were incubated with 10 ug/ml cycloheximide (CHX) for the indicated times. Lysates were harvested and analyzed by western blot. (E) HEK293T cells were transfected with PARP1-Flag for 24 h, and lysates were subjected to immunoprecipitation using an anti-flag antibody and analyzed by western blot with indicated antibodies. The data are presented as the mean ± SD. All $*p < 0.05$, $**p < 0.01$, $***p < 0.001$.

Supplementary Table 1

The sequences of siRNAs targeting PARP1 and RNF146

Name	5'-3'
PARP1-Si-1	GGACCAAGUGUAUGGUCAAdTdT
	UUGACCAUACACUUGGUCCdTdT
PARP1-Si-2	CCGAGAAAUCUCUUACCUCAAdTdT
	UUGAGGUAAGAGAUUUCUCGGdTdT
RNF146-Si-2	GCCAGUAGUGAUAGUGAGGAUdTdT

	AUCCUCACUAUCACUACUGGCdTdT
RNF146-Si-3	CCUGUUCUAAUACUGCACCUUdTdT
	AAGGUGCAGUAUUAGAACAGGdTdT

Supplementary Table 2

The CHIP-qPCR primer sequences were as follows:

Gene	Primer	
DIMT1-P1	Forward	GGGAGCCTACAGATCCCAGA
	Reverse	GACCTCAGCGATCCCAGAAC
DIMT1-P2	Forward	CTAGCGTGAGAAAGCCACCA
	Reverse	CAAGGGCTCCAACTCCAGAC
DIMT1-P3	Forward	CTCTGAGCTCCTCCTCCAGA
	Reverse	GACAAGGGCTCCAACTCCAG

Supplementary Table 3

The qRT-PCR primer sequences were as follows:

Gene	Primer	
DIMT1-1	Forward	GCTGGAGGACTCATGTTCAAC
	Reverse	CCTTGGGTCAAGTTCACAAGC
DIMT1-2	Forward	GACCCAAGGCTAGTAGCTGAA
	Reverse	AGTCGGAGGGCAAATTCTCTT
RNF146	Forward	AAACAGGAAAGCGAACGAGTC
	Reverse	GTTTGCAGACAAATGGCACAT
